# Mesothelial Cells in Fibrosis: Focus on Intercellular Crosstalk

**DOI:** 10.3390/biom16010085

**Published:** 2026-01-05

**Authors:** Nadezhda Bakalenko, Evdokiya Kuznetsova, Konstantin Dergilev, Irina Beloglazova, Anna Malashicheva

**Affiliations:** 1 Institute of Cytology, Russian Academy of Sciences, 194064 St. Petersburg, Russia; 2 Institute of Experimental Cardiology Named after Academician V.N. Smirnov, Federal State Budgetary Institution National Medical Research Center of Cardiology Named after Academician E.I. Chazov, Ministry of Health of the Russian Federation, 121552 Moscow, Russia

**Keywords:** mesothelial cells, fibrosis, mesothelial-to-mesenchymal transition (MMT), cell crosstalk

## Abstract

Mesothelial cells line serosal cavities and internal organs, playing a vital role in maintaining serosal integrity and homeostasis. Their remarkable plasticity and ability to undergo mesothelial-to-mesenchymal transition (MMT) position them as key regulators of tissue repair. However, when normal repair processes fail, mesothelial cells can acquire a profibrotic phenotype. They actively contribute to all stages of fibrosis development, including inflammation, fibrin accumulation, myofibroblast differentiation, and extracellular matrix (ECM) remodeling. Fibrotic progression involves multiple cell types, and communication among them is essential for its perpetuation. Mesothelial cells are implicated in bidirectional crosstalk with fibroblasts, macrophages, lymphocytes, and endothelial cells of the serosal microenvironment through direct contact, paracrine signaling, and extracellular vesicle exchange. These interactions regulate immune cell recruitment, cytokine balance, endothelial permeability, and ECM deposition, while, in turn, immune and endothelial cells modulate mesothelial activation, proliferation, and transition. Understanding this complex network of intercellular communication provides new insights into fibrosis pathogenesis and reveals promising targets for antifibrotic therapies.

## 1. Mesothelial Cells Characteristics and Functions

Mesothelial cells form a continuous monolayer, called the mesothelium, that covers the pleural, peritoneal, and pericardial cavities. Moreover, it is an integral component of the perimetrium, the serous covering of the uterus, and also lines the cavity enclosing the testes [1,2].

Mesothelial cells attach to a thin basement membrane supported by sub-serosal connective tissue. The submesothelial connective tissue layer comprises blood and lymphatic vessels, fibroblasts, mast cells, monocytes/macrophages, leukocytes, adipocytes, and nerve fibers [3,4].

Mesothelial cells display characteristic epithelial features, such as a polygonal shape, apical–basal polarity, and surface microvilli [5,6]. Mesothelial cells express both epithelial (cytokeratins, calretinin, ZO-1) and mesenchymal markers (desmin, vimentin, α-smooth muscle actin). They also express mesothelial markers mesothelin (MSLN), podoplanin (PDPN) and Wilms’ tumor protein 1 (WT1) [3,7,8,9,10,11].

The mesothelium promotes smooth surface movement through the secretion of mucus. Its glycocalyx contains a rich supply of glycosaminoglycans, particularly hyaluronan, which is a highly hydrophilic, anionic disaccharide polymer that forms a hydrated gel-like layer [12].

The mesothelium acts as a semipermeable barrier, separating fluid-filled body cavities from the blood and lymphatic vessels within the underlying submesothelial connective tissue. Through its permeability to electrolytes and fluid, the mesothelial monolayer regulates both secretion and reabsorption in serous cavities, processes largely mediated by transmembrane ion pumps (Na^+^/K^+^-ATPase) and aquaporins [13].

Previously, the mesothelium was thought to have only these two functions. Now it is clear that the mesothelium is a physiologically active tissue with a pivotal role in maintenance of serosal homeostasis [14,15,16].

Mesothelial cells regulate coagulation and fibrinolysis in serosal cavities by secreting both procoagulant and fibrinolytic enzymes [17,18,19].

Mesothelium is an important regulator of inflammation and the immune response. Mesothelial cells secrete proinflammatory cytokines such as interleukins, tumor necrosis factor α (TNF-α), interferon γ, and others [20,21,22]. Mesothelial cells express a variety of receptors, which recognize carbohydrates and lipopolysaccharides on the surface of microbial pathogens such as bacteria, fungi, and viruses and in response to infection release inflammatory mediators that trigger inflammation and activate immunomodulatory pathways [14,23]. The adhesion and migration of various T lymphocytes across the mesothelium monolayer are mediated by different integrins displayed on the surface of mesothelial cells [24]. Mesothelial cells also have antigen-presenting capacity and phagocytic activity. For example, peritoneal mesothelial cells can phagocyte bacteria—Escherichia coli and Staphylococci—as well as dying tumor cells [25,26,27]. Human peritoneal mesothelial cells express major histocompatibility complex class II (MHC II) in response to interferon-γ stimulation and function as effective antigen-presenting cells when exposed to antigens such as tetanus toxoid (TT) or Staphylococcus aureus α-toxin [27,28].

Mesothelial cells produce a wide range of extracellular matrix (ECM) components that are essential for cellular function and the repair of serosal membranes. They synthesize ECM molecules such as collagen types I, III, and IV, elastin, fibronectin, laminin, and proteoglycans, and also contribute to ECM remodeling by releasing matrix metalloproteinases and their tissue inhibitors [29,30,31].

## 2. Mesothelial-to-Mesenchymal Transition (MMT)

Mesothelial cells undergo mesothelial-to-mesenchymal transition (MMT), a process analogous to the epithelial-to-mesenchymal transition (EMT) observed in epithelial cells [32,33]. During this process, mesothelial cells lose cell–cell junctions, reduce their adhesion to the basal lamina, lose apical–basal polarity and acquire a mesenchymal phenotype and enhanced migratory capacity. At the molecular level, this is accompanied by the downregulation of epithelial markers and the upregulation of mesenchymal markers [34,35,36]. This process is regulated by the TGF-β1 pathway, which induces the transcription of numerous mesenchymal genes. TGF-β1 induces MMT in human mesothelial cell cultures isolated from the pleura, omentum, or mesenteric tissue [16,37,38]. In mesothelial cells, TGF-β1 activates both canonical (Smad-dependent) and non-canonical pathways [39], such as the extracellular signal-regulated kinase (ERK) pathway, the c-Jun N-terminal kinase (JNK) pathway, and the p38 mitogen-activated protein kinase (MAPK) pathway. Smad proteins translocate to the nucleus to induce the transcription of MMT-related genes, including SNAI1, SNAI2, SLUG, TWIST1, ZEB1, and ZEB2, which suppress epithelial markers such as E-cadherin and cytokeratins [40,41,42,43]. Numerous studies have demonstrated that Notch signaling plays an essential role in regulating EMT and functionally interacts with the TGF-β1 pathway [44,45,46]. However, research on Notch signaling in mesothelial cells and its involvement in MMT remains limited. In rat peritoneal mesothelial cell cultures, inhibition of the Notch pathway prevents TGF-β1-induced MMT [47]. Similarly, Notch pathway activation has been shown to be essential for the MMT of rat epicardial cells [48].

While TGF-β1 is a well-known inducer of MMT, BMP-7 counteracts this process by directly inhibiting TGF-β–induced Smad-dependent signaling [49,50]. A transcriptomic study revealed that MMT is accompanied by downregulation of genes related to insulin-like growth factor (IGF) and bone morphogenetic protein (BMP) signaling pathways. Treatment with recombinant BMP4 or IGF-binding protein 4 (IGFBP4) was shown to attenuate TGF-β1–induced MMT in cultured cells [34].

Profibrotic cytokines such as TGF-β1 are not the only inducers of MMT. Recent studies show that mechanical cues, including cyclic stretch and alterations in ECM stiffness, also play a crucial role in mesothelial activation. These mechanical signals drive adaptive cellular responses, such as cytoskeletal reorganization and changes in gene expression. The key effectors of the Hippo signaling pathway, transcriptional co-factors Yes-associated protein (YAP) and its paralog WW domain-containing transcription regulator 1 (WWTR1/TAZ), have emerged as central regulators that integrate mechanotransduction with other cellular functions [51]. Research conducted on primary human peritoneal mesothelial cells has shown that cyclic mechanical stretch alone is sufficient to induce bona fide MMT. YAP/TAZ transcriptional activity supports the maintenance of MMT-associated phenotypic changes. In addition, caveolin-1 (CAV1), a mechanosensory protein whose expression in mesothelial cells is regulated by YAP, helps limit MMT by negatively modulating TGF-β1 signaling [52]. Another study demonstrated that mesothelial cells cultured on stiff hydrogels exhibited enhanced nuclear accumulation of TAZ and increased myofibroblastic differentiation. Knockdown of Taz suppressed MMT under these conditions, whereas inhibition of TGF-β1 signaling did not suppress the transition [53]. Taken together, these findings revealed an interaction between biomechanical and biochemical signals in the induction of MMT.

MMT plays a pivotal role in organ development, tissue repair, and the progression of fibrosis [54,55].

## 3. Mesothelial Cells in Tissue Repair

MMT process makes a significant contribution to the development of different organs. For example, many studies of lineage tracing in mice have revealed that, during development, mesothelial cells undergo MMT, migrate and contribute to vascular smooth muscle formation in the gut, heart, liver, and lungs [56,57,58,59].

Many studies suggest that postnatal mesothelial cells retain the differentiative potential of embryonic mesothelium and are capable of giving rise to fibroblasts and vascular smooth muscle cells via MMT [60,61,62]. Moreover, primary rat and human mesothelial cells cultured in osteogenic or adipogenic media undergo MMT and acquire mRNA expression profiles characteristic of osteoblast- and adipocyte-like cells, respectively [63]. Upon liver injury, MCs can migrate inward from the liver surface and produce hepatic stellate cells [64]. The remarkable plasticity and multipotent nature of mesothelial cells suggest their significant contribution to tissue repair and organ regeneration.

Transforming mesothelial cells during MMT secrete a wide range of paracrine factors that influence tissue regeneration. For instance, studies using a transwell coculture system in which mesothelial epicardial cells were grown on a specialized bioceramic scaffold and cardiomyocytes (CMs) were damaged by liposaccharide-induced inflammatory stimulation, demonstrated that epicardial cells on this scaffold undergo Notch-dependent mesothelial-to-mesenchymal transition. During this process, they release paracrine signals that enhance intercellular connectivity among CMs and restore the aberrant expression of cardiac-specific proteins in the damaged CMs [48].

MMT is also initiated in the adult organism in response to mesothelial layer injury. Mesothelial cells near the damaged site lose their junctions, acquire mesenchymal phenotype, proliferate and migrate into the wound. There is evidence that mesothelial cells surrounding the wound make the primary contribution to serosal regeneration. The mesothelium is a slowly proliferating tissue, with only 0.16–0.5% of cells undergoing mitosis at any given time. However, within 48 h of injury, 60–80% of mesothelial cells at the wound edge become mitotically active, while the subserosal cells showed no increase in mitotic activity [65]. Interestingly, mesothelial cells are also thought to detach from adjacent surfaces following injury, freely float in serosal fluid, and subsequently adhere to denuded sites, where they form cellular islands that coalesce to complete re-epithelialization [66,67].

MMT is essential for normal organ development and tissue repair; however, aberrant or sustained activation of this process contributes to pathological conditions such as tumor progression and fibrosis.

## 4. Mesothelial Cells in Fibrosis

Mesothelial cells have been linked to a range of fibrotic disorders, including pulmonary, peritoneal and cardiac fibrosis, and postoperative adhesions.

### 4.1. Pleural Fibrosis

Pleural fibrosis can be caused by a variety of inflammatory conditions, including tuberculous effusion, bacterial empyema, asbestos exposure, rheumatoid pleurisy, cancer, hemorrhagic effusion, and certain medications. Pleural fibrosis can manifest as isolated, localized plaques on the pleura or as a more diffuse form characterized by generalized pleural thickening [68]. The mesothelial cell is a crucial participant in the process of pleural fibrosis, recruiting immune cells to the pleural cavity, secreting proinflammatory cytokines, and regulating coagulation and fibrinolytic pathways [7,16].

### 4.2. Idiopathic Pulmonary Fibrosis

Idiopathic pulmonary fibrosis (IPF) is a progressive disorder characterized by excessive proliferation of connective tissue and abnormal deposition of the extracellular matrix (ECM), leading to irreversible remodeling of lung tissue and impaired respiratory function [69]. Lungs in IPF demonstrate septal thickening, honeycombing, fibroblastic foci, and interstitial inflammation [70]. The pathogenesis of IPF is not yet well understood, although the processes underlying it are thought to be similar to those seen in other types of tissue fibrosis. A crucial aspect in the progression of fibrosis is the accumulation of myofibroblasts within lung tissues. Myofibroblasts produce ECM and release fibrogenic cytokines. The precise origins of myofibroblasts in lung fibrosis remain uncertain. Myofibroblasts can arise from lung stromal cells, including resident fibroblasts and pericytes, from mesenchymal stem cells in the bone marrow, and through the process of transdifferentiation of alveolar epithelial cells and pleural mesothelial cells [7,45]. Pleural mesothelial cells can undergo MMT and transform into myofibroblasts. Transformed pleural mesothelial cells were identified within the lung parenchyma of patients with IPF. They also secrete fibrogenic factors that promote fibroblast activation [71].

### 4.3. Peritoneal Fibrosis Caused by Peritoneal Dialysis

Peritoneal dialysis (PD) is a valuable method of renal replacement therapy for patients with end-stage renal disease. However, a significant limitation of this approach is that the fluids used for PD often lead to peritoneal fibrosis, which develops in most patients within the first two years of treatment [16]. In peritoneal biopsies taken from patients with PD, significant changes in the structure of the mesothelium are found: mesothelial cells increase in size, contain fewer microvilli, and have weak adhesion [72]. During PD, the mesothelial cells lining the peritoneum are exposed to a hypertonic environment with high levels of glucose and glucose degradation products, which causes decrease in mesothelial cell viability and disruption of their normal function. High levels of glucose react with long-lived proteins, such as collagen, to form advanced glycation end products (AGEs). AGE-modified collagen becomes more rigid and less prone to enzymatic breakdown, which in turn contributes to the accumulation of ECM proteins [73]. Mesothelial damage also leads to the recruitment of immune cells, initiates inflammatory response and ultimately drives the progression of fibrosis [16].

### 4.4. Postoperative Adhesions

Intra-abdominal and pelvic adhesion formation is a very common postoperative complication. They can cause chronic pelvic pain, intestinal obstruction, and infertility. Surgical injury to the serosa provokes an inflammatory response and disrupts normal fibrin turnover. Reduced secretion of fibrinolytic factors by mesothelial cells leads to the formation of fibrin bands between tissues and organs, which subsequently mature into fibrous adhesions [15].

### 4.5. Cardiac Fibrosis

Cardiac fibrosis develops in response to various stresses, including myocardial infarction (MI), hypertensive heart disease, diabetes, and aging. It is marked by excessive ECM deposition, which increases stiffness and impairs cardiac function. The origin and activation of cardiac fibroblasts depend on the type of cardiac stress. Epicardial cells play a significant role in some types of cardiac injury, such as MI. Recent studies have shown that the embryonic epicardium is the main source of cardiac fibroblasts, and this developmental process can be reactivated in the adult heart during the fibrotic response [74].

### 4.6. Molecular Mechanisms of Fibrosis and Mesothelial Cell Contribution

Fibrosis development encompasses inflammation, abnormal fibrin accumulation and fibrin mesh formation, myofibroblast differentiation, and excessive extracellular matrix (ECM) deposition [75,76]. Mesothelial cells are actively involved in all these processes [16].

Synthesis and degradation of extracellular matrix components are balanced by matrix metalloproteinases (MMPs) and their inhibitors (TIMPs). Mesothelial cells secrete both MMPs and TIMPs, maintaining ECM homeostasis [77]. Numerous studies have shown that, in response to inflammatory cytokines, the expression of these proteins is altered, disrupting the MMP/TIMP balance and leading to dysregulated ECM turnover. The specific MMP and TIMP components affected vary depending on the cellular context and type of stimulus [30,78,79,80].

Fibrin deposition is an early step in normal wound repair, but when this provisional fibrin-rich matrix persists, it acts as a scaffold for tissue repair cell ingrowth and leads to increased collagen accumulation and fibrosis [16]. Mesothelial cells are key regulators of the balance between fibrin synthesis and degradation in serosal cavities. They express both pro-coagulation factors such as tissue factor (TF), plasminogen activator inhibitors (PAI) 1 and 2, and fibrinolytic mediators, such as TF pathway inhibitor (TFPI), tissue plasminogen activator (tPA), urokinase PA (uPA), and uPA receptor (uPAR) [19,81,82,83]. Pro-fibrotic stimuli and inflammation increase PAI-1 expression and reduce tPA production by mesothelial cells, promoting fibrin deposition [84]. Moreover, PAI-1 can bind to tPA and act as a chemokine, attracting macrophages. These macrophages, in turn, further enhance PAI-1 secretion by upregulating the HER1 receptor on peritoneal mesothelial cells, thereby amplifying fibrin accumulation [85].

The principal effector cells responsible for extracellular matrix (ECM) synthesis during fibrogenesis are myofibroblasts, which produce large amounts of structural proteins such as collagens I, III, IV and fibronectin [86,87]. Myofibroblasts originate from multiple cellular sources, predominantly from resident fibroblasts [88]; however, mesothelial cells can also undergo MMT and contribute significantly to the myofibroblast population. In vitro studies reveal that TGF-β1 induces pleural mesothelial cells and peritoneal mesothelial cells transformation into myofibroblasts [37,89]. The profibrotic influence of TGF-β1 can be mediated, at least in part, through the Notch signaling pathway. For example, inhibition of Notch signaling was shown to block TGF-β1-induced expression of α-smooth muscle actin (α-SMA) and collagen I in cultured rat peritoneal mesothelial cells. Moreover, in vivo experiments demonstrated that intraperitoneal administration of the γ-secretase inhibitor DAPT, which prevents Notch activation, significantly attenuated peritoneal fibrosis [47]. Adult epicardial mesothelial cells can also undergo MMT upon cardiac stress, such as hypertension or infarction, to generate myofibroblast-like cells [90]. The migration of pleural mesothelial cells into the lung parenchyma and their differentiation into myofibroblasts have been demonstrated in vivo using a mouse model [71,91]. Lineage-tracing study in a mouse model of peritoneal fibrosis induced by chlorhexidine gluconate demonstrated that 17% of myofibroblasts within the fibrotic connective tissue were derived from mesothelial cells [92].

Although lineage-tracing studies support a contribution of mesothelial cells to fibroblast and myofibroblast populations during fibrosis, these approaches have important limitations. Commonly used drivers such as WT1 lack absolute specificity, as WT1 expression can overlap with other mesenchymal populations and may be reactivated upon injury, potentially confounding fate-mapping results (e.g., Wt1^CreERT2 models) [93,94]. Moreover, recombination efficiency, timing of induction, and injury context vary substantially across studies, leading to divergent quantitative estimates of MMT–derived cells.

New research, based on single-cell transcriptomic compendiums of mesothelium across healthy and fibrotic mouse and human organs, revealed mesothelial cells transition through no less than four different cell states during fibrosis. In bleomycin-induced fibrosis model, mesothelial cells initially differentiate into a metabolically active phenotype (i). After several days, this population gains a proteolytic phenotype (ii), which then transitions into an immune-modulatory phenotype (iii), before finally adopting a fibrogenic phenotype (iv). Each phenotype plays a different role in the pathogenesis of lung fibrosis [95].

## 5. Mesothelial Crosstalk in Fibrosis

Interactions between mesothelial cells and submesothelial stromal cells are crucial for maintaining normal tissue homeostasis and for the development of fibrosis. In particular, crosstalk with immune cells and fibroblasts plays a pivotal role in these processes.

### 5.1. Interplay Between Mesothelial Cells and Fibroblasts

Many studies have demonstrated that injured mesothelial cells secrete a wide range of molecules that influence stromal fibroblasts. Fibroblasts are widely recognized as the main precursors of myofibroblasts during fibrotic progression [96,97,98,99].

An important property of the mesothelium is its ability to produce TGF-β1, the key master regulator of fibrosis [100]. Mesothelial cells have been shown to release substantial amounts of TGF-β1 in response to various stimuli [7,101,102,103]. TGF-β1 acts as a potent chemoattractant for fibroblasts, promotes their migration and differentiation into myofibroblasts, and enhances the synthesis of extracellular matrix components [104,105,106,107].

Mesothelial cells can also release PDGF, which drives fibroblast proliferation by overriding G1 cell cycle checkpoints and exerts pleiotropic effects, including chemotaxis and stimulation of extracellular matrix and collagen synthesis [108,109]. Subserosal peritoneal fibroblasts have been shown to express platelet-derived growth factor receptor-α (PDGFRA), and pharmacological inhibition of this receptor attenuates peritoneal fibrosis [110]. In mouse models, PDGF has been shown to mediate fibroblast proliferation in the pleura following exposure to inhaled crocidolite asbestos fibers, whereas neutralizing antibodies against PDGF suppress this proliferative response [111]. Moreover, PDGF induces the expression of TGF-β, thereby amplifying the fibrotic cascade [112].

Basic fibroblast growth factor (bFGF, also known as FGF-2) stimulates fibroblast proliferation and migration. Pleural mesothelial cells secrete bFGF, and its production increases in the presence of sclerosants. Several studies suggest that bFGF levels are significant for pleural fibrosis; notably, elevated pleural fluid concentrations of bFGF are associated with successful pleurodesis, whereas lower levels correlate with pleurodesis failure [7,17,113].

The direct influence of mesothelial cells on the profibrotic transformation of fibroblasts has been demonstrated in several in vitro experiments.

More than two decades ago, Antony et al. reported that conditioned media from talc-stimulated pleural mesothelial cells induced a twofold increase in fibroblast proliferation. The addition of anti-bFGF antibody caused a significant reduction (30 to 40%) in fibroblast growth, indicating a bFGF-specific response [113].

Later studies provided a more detailed analysis of mesothelial–fibroblast interactions using conditioned media experiments. Pulmonary fibroblasts cultured in medium derived from pleural mesothelial cells showed slightly increased expression of key fibrotic markers, such as collagen I and α-SMA. Conditioned medium from bleomycin-stimulated pleural mesothelial cells induced a marked upregulation of these markers in lung fibroblasts. Interestingly, while direct exposure of pulmonary fibroblasts to bleomycin inhibited their proliferation, supernatant from bleomycin-activated mesothelial cells instead promoted fibroblast proliferation. These data suggest that activated pleural mesothelial cells secrete profibrotic factors that enhance collagen I synthesis and promote the transdifferentiation of lung fibroblasts into myofibroblasts [91]. Fibroblast-conditioned medium also induced MMT in pleural mesothelial cells, underscoring the bidirectional crosstalk between mesothelial cells and fibroblasts. Furthermore, it has been shown that the underlying mechanism involves signaling pathways mediated by TGF-β1, Wnt/β-catenin, and CD147. Blockage of any of these signalings attenuates culture-medium-induced fibrotic transformation [91].

Activated peritoneal mesothelial cells are also capable of inducing profibrotic transformation in peritoneal fibroblasts. In a study by Wei and colleagues, mesothelial cells were activated using a glucose degradation product methylglyoxal (MGO). Glucose degradation products are considered as key contributors to the development of peritoneal fibrosis. Transcriptomic analysis of mesothelial cells after incubation with MGO revealed upregulation of proinflammatory and profibrotic genes. Peritoneal fibroblasts exposed directly to MGO exhibit no detectable response; however, conditioned medium from MGO-stimulated mesothelial cells induced fibroblast transformation into proto-myofibroblasts, activated fibroblasts in the first stage toward myofibroblasts. This transformation was not fully completed, leading the authors to suggest that additional factors or cell types (e.g., macrophages) may be required to drive full differentiation into myofibroblasts [114].

In addition to paracrine factors, peritoneal mesothelial cells also produce extracellular vesicles (EVs). Extracellular vesicles (EVs) are increasingly recognized as key mediators of intercellular communication [115]. Growing evidence indicates that peritoneal cells release diverse EVs into PD effluent in response to bioincompatible dialysate exposure [116]. EVs isolated from peritoneal dialysis (PD) effluent were analyzed by integrative EVs proteomics and single-cell RNA sequencing, revealing mesothelial cells as the main EVs source. In vitro, TGF-β1 stimulates mesothelial cells to release EVs, which in turn promote fibroblast activation and peritoneal fibrogenesis. Inhibition of EV secretion by either GW4869 or Rab27a knockdown markedly suppresses PD-induced fibroblast activation and fibrosis. Mechanistically, injured mesothelial cells release EVs enriched in integrin-linked kinase (ILK), which are transferred to fibroblasts and activate them via the p38 MAPK pathway [117].

There is growing evidence that fibrotic ECM produced by myofibroblasts is not merely a hallmark and effector of fibrosis but also a key driver of its progression. In the absence of exogenous cytokines, decellularized lung ECM from patients with IPF is sufficient to induce the transformation of normal lung fibroblasts into myofibroblasts [118]. Increased ECM stiffness activates YAP, the mechanosensitive effector of the Hippo pathway, in adjacent fibroblasts and other cell types, which in turn enhances ECM production and further increases ECM stiffness, creating a positive feedback loop [119]. This profibrotic ECM can also interact with mesothelial cells, promoting MMT through activation of the YAP/TAZ pathway [52,53].

**Figure 1:**
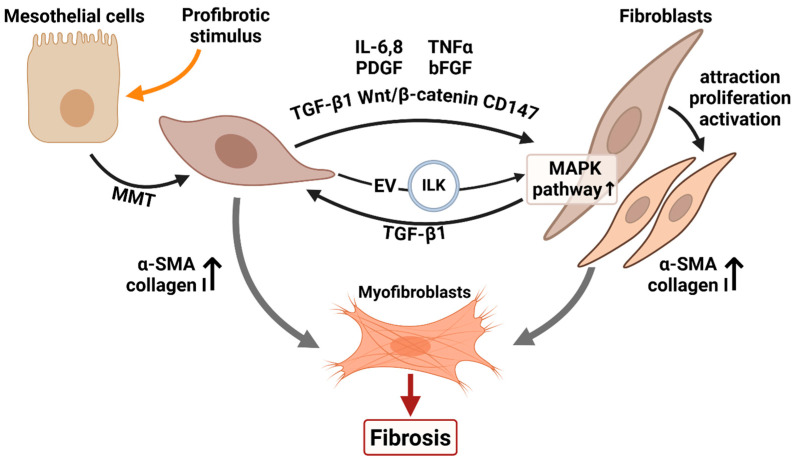
Mesothelial-fibroblast communication in fibrogenesis. In response to profibrotic stimuli, mesothelial cells undergo MMT and migrate into the submesothelial stroma, where they may differentiate into myofibroblasts. Activated mesothelial cells secrete cytokines and extracellular vesicles that promote the recruitment, proliferation, and activation of fibroblasts, driving their conversion into myofibroblasts. These myofibroblasts synthesize extracellular matrix, ultimately leading to the development of fibrosis. MMT—mesothelial-to-mesenchymal transition, IL—interleukin, TNFα—tumor necrosis factor alpha, PDGF—platelet-derived growth factor, bFGF—basic fibroblast growth factor, EV—extracellular vesicle, TGF-β1—transforming growth factor beta 1, α-SMA—alpha-smooth muscle actin. “Created with BioRender.com”.

Mesothelial–fibroblast communication is pivotal for fibrosis development, operating through pathways such as TGF-β1, p38 MAPK, Wnt, and CD147, and mediated by both soluble factors and extracellular vesicles (Figure 1).

### 5.2. Communication Between Mesothelial and Immune Cells

Inflammation is a key driver of fibrosis, highlighting the critical role of immune cells in fibrotic processes. Mesothelial cells are increasingly recognized as active regulators of inflammation and immune cells recruitment [7,120,121,122].

When the serosa is exposed to infection or injurious agents such as dialysis fluid or asbestos, a massive influx of leukocytes occurs from the vasculature into the serosal space [23,72]. Mediators released by activated macrophages, including TNF-α, IL-1β, and interferon-γ (IFN-γ), stimulate mesothelial cells to produce cytokines. Notably, in vitro experiments have demonstrated that mesothelial cells can produce pro-inflammatory cytokines in response to various noxious stimuli, even in the absence of immune cells, suggesting that mesothelial cells may represent the first link in the initiation of the inflammatory response [21,123,124].

The molecular mechanisms underlying the inflammatory response of mesothelial cells are extensively studied. Cellular stress and tissue injury lead to the generation of extracellular matrix (ECM) degradation products such as hyaluronan, biglycan, and fibronectin, and the release of endogenous cellular components, such as mitochondrial reactive oxygen species (ROS) and mitochondrial DNA, collectively known as damage-associated molecular patterns (DAMPs). These DAMPs are recognized by Toll-like receptors (TLRs), specifically TLR2 and TLR4, on mesothelial cells. TLRs activation induces the production of the inflammatory cytokines [120,125]. Inhibiting TLR-signaling using soluble TLR2 attenuates inflammation [126]. Cellular stress stimulates mesothelial cells to release high mobility group box-1 (HMGB1) protein from the nuclei. HMGB1 acts on mesothelial cells as an autocrine factor, promoting cytokine expression via activation of the mitogen-activated protein kinase (MAPK) signaling pathway [120,127]. NF-κB and JAK/STAT signaling pathways are also critical regulators of the mesothelial inflammatory response [128,129,130].

Mesothelial cells are capable of secreting a wide range of cytokines such as TNF-α, IL-6, IL-8, IL-1β, MCP-1, IL-17, IL-22 and others [123,131,132]. The profile of cytokines and their expression levels vary according to the specific stimulus [132]. These cytokines, released by mesothelial cells, attract various immune cells—including monocytes, macrophages, leukocytes, and neutrophils—to the site of injury. A special role is played by IL-6 trans-signaling, in which IL-6 forms a complex with its soluble receptor (sIL-6R). This complex activates cells lacking the membrane-bound receptor, such as mesothelial cells and fibroblasts, initiating JAK/STAT signaling and creating a chronic positive feedback loop that amplifies inflammation and fibrosis [133,134].

Mesothelial crosstalk with macrophages plays a pivotal role in the development of inflammation and the initiation of fibrosis. Monocytes and macrophages constitute a significant proportion (50–90%) of the infiltrated leukocytes in peritoneal and pleural cavities [135,136]. Macrophages can generally be classified into tissue-resident and monocyte-derived populations. While monocyte-derived macrophages are primarily associated with the progression of inflammation and fibrosis, tissue-resident macrophages (TRM) play a role in maintaining tissue homeostasis [120]. The proliferation and survival of peritoneal TRMs depend on CSF1. Mesothelial cells produce both soluble and membrane-bound forms of CSF1, which together sustain the growth and maintenance of peritoneal macrophages [137]. Peritoneal mesothelial cells play a crucial role in the differentiation and maintenance of a specific subpopulation of GATA6-expressing macrophages within the peritoneal cavity. These macrophages are essential for pathogen defense and tissue repair. Soluble proteins such as mesothelin (MSLN) and mucin 16 (MUC16), produced by mesothelial cells, are required to sustain GATA6 expression in resident peritoneal macrophages, thereby preserving their phenotype and functional identity [138]. Following injury, GATA6^+^ macrophages rapidly migrate into damaged areas through direct recruitment across the mesothelium. This invasion depends on the binding of macrophage CD44 to exposed mesothelial hyaluronan at the injury site [139]. Pleural mesothelial cells and resident pulmonary macrophages both express the adhesion molecules CD54 and CD11a. These molecules mediate mesothelial–macrophage interactions, leading to the detachment of mesothelial cells. This detachment represents an initial step in the formation of pleural adhesions [140].

Cytokines and chemokines produced by mesothelial cells promote the infiltration of monocytes and monocyte-derived macrophages into sites of injury. In addition to soluble chemoattractants, direct interactions with the mesothelium are also essential for macrophage recruitment. Pro-inflammatory stimuli upregulate ICAM-1 expression on the surface of pleural mesothelial cells, which plays a pivotal role in facilitating macrophage adhesion and migration [141]. Macrophage emigration from the site of inflammation into the lymphatics is also regulated by mesothelial cells. Macrophages adhere specifically to the mesothelium overlying draining lymphatics, and their emigration rate is regulated by the macrophage activation state. This adhesion is Arg-Gly-Asp (RGD)–sensitive and partially mediated by very late antigen VLA-4 and VLA-5 integrins. Furthermore, macrophage clearance into lymphatics can be inhibited in vivo by RGD peptides as well as by blocking VLA-4 and VLA-5 [142].

Monocyte-derived macrophages undergo polarization into two major phenotypes—M1 and M2. M1 macrophages are primarily associated with pro-inflammatory responses and pathogen defense, whereas M2 macrophages are involved in debris clearance, tissue repair, and fibrosis development [46]. Factors produced by mesothelial cells can affect macrophage polarization and M1/M2 proportion [143].

Macrophages, in turn, influence mesothelial cell behavior by promoting MMT, modulating their proliferation, and enhancing collagen production [144,145]. Co-cultivation of peritoneal mesothelial cells with M1 macrophages induces phenotypic changes, down-regulation of E-cadherin expression and increasing α-SMA levels, indicating that M1 macrophages facilitate MMT in mesothelial cells. In contrast, mesothelial cells co-cultured with M2 macrophages show no significant alterations [145]. Another study, however, has shown different results with the same peritoneal mesothelial cell line. Co-culture experiments with polarized macrophages (M1, M2a, M2c) revealed that all subtypes induced MMT in PMCs, characterized by reduced E-cadherin and increased α-SMA expression. These effects were most pronounced with M2c macrophages, suggesting their stronger profibrotic influence [146].

Macrophage-mesothelial cell crosstalk forms a positive feedback loop whereby direct interaction with CX3CR1-expressing macrophages promotes mesothelial expression of CX3CL1 and TGF-β1 expression. In turn, TGF-β1 upregulates CX3CR1 in murine and human monocytic cells. It is interesting that only direct co-culture with macrophages induced TGF-β and CX3CL1 mRNA expression in mesothelial cells. The increase in TGF-β1 and CX3CL1 was completely abolished in transwells [122].

Under specific stimuli, mesothelial cells differentiate into phagocytic (macrophage-like) cells, expressing macrophage markers (OX43, ED1, CD68), which indicates a potential for transdifferentiation into macrophage-like cells [147,148]. Granulocyte–macrophage colony-stimulating factor (GM-CSF), produced by the mesothelial cells, probably has an autocrine regulatory role in this transition [149,150].

T-lymphocyte contribution to inflammation and fibrosis development also depends on their crosstalk with the mesothelium. T-cell recruitment to the peritoneal cavity during inflammation requires adhesion to mesothelial cells via integrins α6β1 and α4β1. Blocking α6β1 inhibited Th1 migration, while anti-α4 antibody reduced Th2 migration across mesothelial monolayers, indicating that integrins mediate selective adhesion and migration of T-cell subsets through the mesothelium [24].

Inflammatory stimuli induce the expression of ICAM-1 and VCAM-1 in pleural mesothelial cells. These adhesion molecules regulate selective CD4+ T cells adhesion, activation, and expansion, as demonstrated by experiments where pretreatment with anti–ICAM-1 or anti–VCAM-1 antibodies inhibited these processes [151].

Activated CD4^+^ T lymphocytes secrete IL-17A, which stimulates the granulocyte colony-stimulating factor (G-CSF)–NF-κB signaling axis in mesothelial cells. This activation leads to the release of a set of cytokines that promote selective recruitment of polymorphonuclear leukocytes [152,153].

Another subset of CD4^+^ T cells—regulatory T (Treg) cells and T helper 17 (Th17) cells—also contribute to inflammation and fibrosis [154]. Th17 cells secrete large amounts of proinflammatory cytokines, primarily IL-17, and are implicated in numerous chronic inflammatory diseases. In contrast, Treg cells suppress immune responses and maintain immune tolerance [155]. The balance between Th17 and Treg cells is tightly regulated by the surrounding cytokine milieu, suggesting that mesothelial cells, through their diverse cytokine secretion, can influence this ratio [156,157]. In turn, IL-17 produced by Th17 cells can stimulate mesothelial inflammation and promote fibrosis progression [158,159].

While activated mesothelial cells promote lymphocyte recruitment and inflammation, the intact mesothelium appears to have immunosuppressive and anti-inflammatory properties. The secretome of omental mesothelial cells has been shown to suppress mouse and human lymphocyte proliferation, inhibit activated CD19^+^/CD25^+^ B cells, and drive macrophage polarization toward an M2 anti-inflammatory phenotype [160,161,162].

Epicardial mesothelial cells have been shown to recruit regulatory T (Treg) cells to the myocardium, thereby limiting inflammation during acute myocardial infarction. This immunomodulatory function of the epicardium critically depends on Hippo signaling [163].

**Figure 2:**
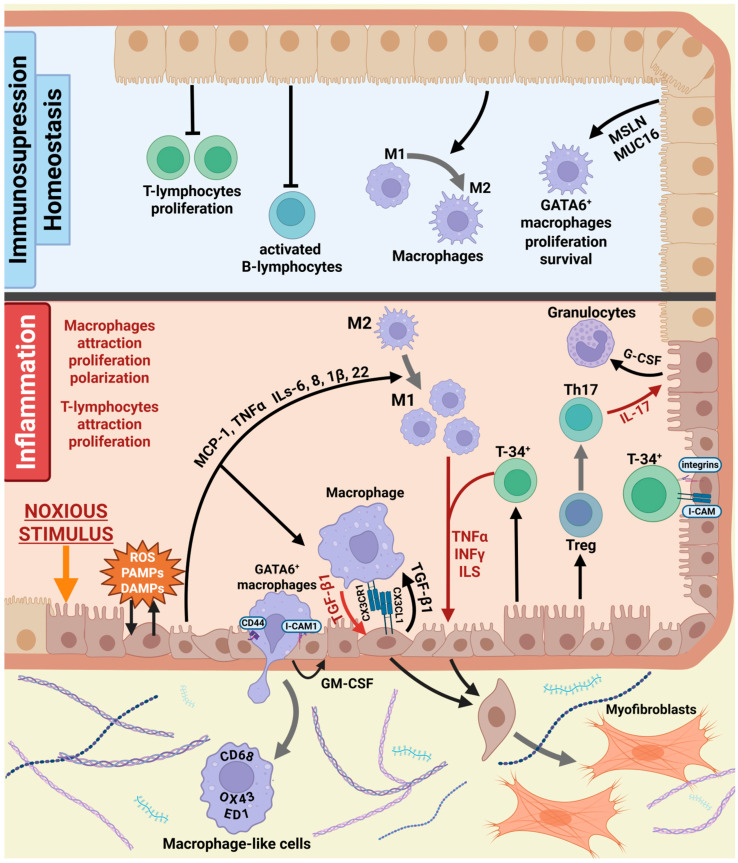
Mesothelial-immune cells interactions. In the absence of injury or profibrotic stimuli, mesothelial cells express factors that support the maintenance of M2 macrophages and inhibit activated B lymphocytes and T-cell proliferation, thereby contributing to overall immune homeostasis. Noxious stimuli that damage the mesothelial layer lead to the release of DAMPs and ROS. These molecules induce mesothelial cells to produce pro-inflammatory cytokines that recruit immune cells and promote their proliferation and activation. Communication occurs both through secreted mediators and through direct contact between immune and mesothelial cells via various adhesion molecules. Immune cells, in turn, sustain the inflammatory response and secrete factors that promote MMT in mesothelial cells. M1—M1 subtype macrophage, M2—M2 subtype macrophage, T-34+—T-lymphocyte CD34+, Th17—T helper CD17+, Treg—regulatory T-lymphocyte, DAMPs—damage-associated molecular patterns, PAMPs—pathogen-associated molecular patterns, ROS—reactive oxygen species, IL—interleukin, TNFα—tumor necrosis factor alpha, INFγ—interferon gamma, I-CAM—immunoglobulin-like cell adhesion molecule, TGF-β1—transforming growth factor beta 1, G-CSF—granulocyte colony stimulating factor, GM-CSF—granulocyte-monocyte colony stimulating factor, CX3CL1—C-X3-C Motif Chemokine Ligand 1, CX3CR1 –CX3CL1 receptor 1. “Created with BioRender.com”.

Mesothelial and immune cells engage in dynamic bidirectional communication that plays a crucial role in the development of fibrosis. Mesothelial cells release factors that attract and activate immune cells such as macrophages, neutrophils, and T lymphocytes. Mesothelial cells regulate the recruitment of specific immune cell subsets through the expression of different adhesion molecules. In turn, activated immune cells influence mesothelial cell behavior, promoting MMT, extracellular matrix deposition, and chronic inflammation, leading to fibrosis progression [7,120,164,165] (Figure 2).

### 5.3. Crosstalk Between Mesothelial and Endothelial Cells

The submesothelial serosal compartment is rich in blood vessels, placing mesothelial and endothelial monolayers in close proximity. Although their crosstalk remains poorly explored, accumulating evidence suggests that interactions between these cell types play an important role in molecular transport, immune responses, angiogenesis, and fibrotic remodeling during injury and inflammation [166].

Neoangiogenesis expands the vascular surface area and enhances solute transport, while simultaneously promoting fibrosis and inflammatory tissue remodeling in peritoneal fibrosis caused by peritoneal dialysis [167]. The alterations of microvessels are also involved in IPF. These can be one of the factors leading to the development of pulmonary hypertension [168].

Noxious stimuli induce MMT in mesothelial cells, leading to the expression of various factors that influence endothelial cell behavior and function, including angiogenic factors such as VEGF and endothelin-1 [38,169,170]. VEGF plays a particularly important role in promoting endothelial cell proliferation and survival, while also increasing vascular permeability [171,172]. In vitro experiments have shown that peritoneal mesothelial cells activated by glucose and its metabolites enhance their VEGF secretion and increase capillary tube formation by human umbilical vein endothelial cells (HUVEC) [173]. Activated mesothelial cells express proinflammatory cytokines, including IL-β1 and TNF-α [123]. These cytokines were shown to enhance the expression of adhesion molecules such as I-CAM, V-CAM and E-selectin on the endothelial cell surface [174,175]. This facilitates the recruitment of immune cells from the bloodstream to the site of injury, thereby amplifying the inflammatory response [176].

EVs–mediated communication may also be an important mechanism of mesothelial–endothelial interaction during fibrosis. Pro-fibrotic stimuli significantly increase EV generation from human peritoneal mesothelial cells. These EVs prime mesothelial cells toward a profibrotic phenotype and enhance tube formation in endothelial cells. These findings suggest that mesothelial cell-derived EVs actively contribute to both mesothelial activation and angiogenic responses, thereby reinforcing fibrotic progression [177].

Endothelial cells can also modulate mesothelial behavior. Using an in vitro 3D peritoneal diffusion model, it has been demonstrated that endothelial cells affect mesothelial cell permeability and inhibit MMT [178].

At present, evidence describing mesothelial–endothelial crosstalk remains very scarce. in vitro systems based on semi-permeable culture inserts could provide a useful initial platform to elucidate fundamental modes of interaction between these cell types. Subsequently, more advanced three-dimensional culture models may offer improved representation of transport dynamics and structural complexity within the serosal membranes [166].

## 6. Conclusions

The evidence synthesized in this review firmly establishes the mesothelial cell not as a passive bystander but as a central orchestrator of fibrotic pathogenesis. Its remarkable plasticity, epitomized by the MMT, and its extensive, dynamic crosstalk with the stromal microenvironment are fundamental to all stages of fibrosis—from the initiation of inflammation and fibrin deposition to the activation of myofibroblasts and the final, pathological scarring of tissue.

The cumulative message of this review can be distilled into several key takeaways.

MMT is a Multifaceted Process: Driven by a complex interplay of biochemical (e.g., TGF-β, Notch) and biomechanical (e.g., ECM stiffness, YAP/TAZ) signals, MMT is a critical source of profibrotic myofibroblasts.

Fibrosis progression is not a linear cascade but a self-amplifying network. Mesothelial cells activate fibroblasts and recruit/educate immune cells through paracrine signals, extracellular vesicles, and direct contact. In turn, these activated partners (macrophages, fibroblasts, T-cells) feed back to further promote mesothelial activation and MMT, creating a vicious cycle.

The ECM is an Active Player: The fibrotic ECM is not merely a scar but a key driver of disease, promoting MMT and fibroblast activation through mechanosensitive pathways like YAP/TAZ, thereby establishing a positive feedback loop that maintains fibrotic progression.

Pleiotropic Immune Regulation: Mesothelial cells exhibit a dual role in immunity, maintaining homeostasis under normal conditions but driving robust inflammation upon injury. Their specific interactions with different macrophage subsets (M1, M2) and T-cell populations (Th17, Treg) critically shape the balance between resolution and chronic, fibrotic inflammation.

Despite these advances, significant knowledge gaps remain. The precise molecular switches that determine whether mesothelial activation leads to regenerative repair or progressive fibrosis are still unclear. The relative contributions of mesothelial-derived myofibroblasts versus other sources in different organs and disease etiologies need further quantification in human patients. Furthermore, the functional heterogeneity of mesothelial cells across different serosal cavities and their distinct states during fibrosis, as revealed by recent single-cell transcriptomic studies, warrants deeper investigation.

To bridge these gaps and advance the field, we propose a model that suggests that an initial injury (e.g., dialysis fluid, infection, surgery) directly damages the mesothelium, triggering MMT and the release of pro-fibrotic and pro-inflammatory mediators. These signals activate submesothelial fibroblasts and recruit/prime immune cells. The activated fibroblasts produce a stiff, profibrotic ECM, while the immune cells (particularly M2 macrophages and Th17 cells) release copious amounts of TGF-β and other cytokines. This altered microenvironment then acts back on the mesothelium, reinforcing MMT and locking the system into a self-sustaining, feed-forward loop of cellular crosstalk that drives unabated ECM deposition.

This model underscores that therapeutic strategies must move beyond targeting a single cytokine and instead focus on disrupting this integrated network. Future research should prioritize the development of advanced 3D co-culture systems and organ-on-chip models that recapitulate the architecture and mechanical forces of the serosal microenvironment. These tools will be invaluable for dissecting the precise molecular pathways of the Reciprocal Activation Loop and for screening novel interventions that can disrupt this vicious cycle at multiple nodes—for instance, by inhibiting specific EV cargo, modulating mechanosignaling (YAP/TAZ), or re-educating the immune response. A comprehensive understanding of this cellular network will ultimately unlock novel therapeutic strategies to prevent, halt, or even reverse fibrotic diseases.

## Data Availability

No new data were created or analyzed in this study.

## References

[1] Karl J., Capel B. (1998). Sertoli Cells of the Mouse Testis Originate from the Coelomic Epithelium. Dev. Biol..

[2] Whitaker D., Papadimitriou J.M., Walters M.N.-I. (1980). The Mesothelium: A Histochemical Study of Resting Mesothelial Cells. J. Pathol..

[3] Mutsaers S.E. (2004). The Mesothelial Cell. Int. J. Biochem. Cell Biol..

[4] Kawanishi K. (2016). Diverse Properties of the Mesothelial Cells in Health and Disease. Pleura Peritoneum.

[5] Mutsaers S.E. (2002). Mesothelial Cells: Their Structure, Function and Role in Serosal Repair. Respirology.

[6] Mutsaers S.E., Wilkosz S. (2007). Structure and Function of Mesothelial Cells. Cancer Treat. Res..

[7] Batra H., Antony V.B. (2015). Pleural Mesothelial Cells in Pleural and Lung Diseases. J. Thorac. Dis..

[8] Metelmann I.B., Kraemer S., Steinert M., Langer S., Stock P., Kurow O. (2022). Novel 3D Organotypic Co-Culture Model of Pleura. PLoS ONE.

[9] Hagiwara Y., Hamada Y., Kuwahara M., Maeda M., Segawa T., Ishikawa K., Hino O. (2008). Establishment of a Novel Specific ELISA System for Rat N- and C-ERC/Mesothelin. Rat ERC/Mesothelin in the Body Fluids of Mice Bearing Mesothelioma. Cancer Sci..

[10] Jiang L., Yamashita Y., Toyokuni S. (2010). A Novel Method for Efficient Collection of Normal Mesothelial Cells In Vivo. J. Clin. Biochem. Nutr..

[11] Gulyás M., Hjerpe A. (2003). Proteoglycans and WT1 as Markers for Distinguishing Adenocarcinoma, Epithelioid Mesothelioma, and Benign Mesothelium. J. Pathol..

[12] Yung S., Chan T.M. (2007). Hyaluronan—Regulator and Initiator of Peritoneal Inflammation and Remodeling. Int. J. Artif. Organs.

[13] Ji H.L., Nie H.G. (2008). Electrolyte and Fluid Transport in Mesothelial Cells. J. Epithel. Biol. Pharmacol..

[14] Mutsaers S.E., Prêle C.M.A., Pengelly S., Herrick S.E. (2016). Mesothelial Cells and Peritoneal Homeostasis. Fertil. Steril..

[15] Herrick S.E., Wilm B. (2021). Post-Surgical Peritoneal Scarring and Key Molecular Mechanisms. Biomolecules.

[16] Mutsaers S.E., Birnie K., Lansley S., Herrick S.E., Lim C.B., Prêle C.M. (2015). Mesothelial Cells in Tissue Repair and Fibrosis. Front. Pharmacol..

[17] Mierzejewski M., Korczynski P., Krenke R., Janssen J.P. (2019). Chemical Pleurodesis—A Review of Mechanisms Involved in Pleural Space Obliteration. Respir. Res..

[18] Rougier J.P., Guia S., Hagège J., Nguyen G., Ronco P.M. (1998). PAI-1 Secretion and Matrix Deposition in Human Peritoneal Mesothelial Cell Cultures: Transcriptional Regulation by TGF-Β1. Kidney Int..

[19] Bottles K.D., Laszik Z., Morrissey J.H., Kinasewitz G.T. (1997). Tissue Factor Expression in Mesothelial Cells: Induction Both In Vivo and In Vitro. Am. J. Respir. Cell Mol. Biol..

[20] Lüdemann W.M., Heide D., Kihm L., Zeier M., Scheurich P., Schwenger V., Ranzinger J. (2017). TNF Signaling in Peritoneal Mesothelial Cells: Pivotal Role of Cflip_L_. Perit. Dial. Int..

[21] Jonjić N., Peri G., Bernasconi S., Sciacca F.L., Colotta F., Pelicci P., Lanfrancone L., Mantovani A. (1992). Expression of Adhesion Molecules and Chemotactic Cytokines in Cultured Human Mesothelial Cells. J. Exp. Med..

[22] Acencio M.M.P., Soares B., Marchi E., Silva C.S.R., Teixeira L.R., Broaddus V.C. (2015). Inflammatory Cytokines Contribute to Asbestos-Induced Injury of Mesothelial Cells. Lung.

[23] Jantz M.A., Antony V.B. (2008). Pathophysiology of the Pleura. Respiration.

[24] Wang H.H., Lee T.Y., Lin C.Y. (2008). Integrins Mediate Adherence and Migration of T Lymphocytes on Human Peritoneal Mesothelial Cells. Kidney Int..

[25] Visser C.E., Brouwer-Steenbergen J.J., Schadee-Eestermans I.L., Meijer S., Krediet R.T., Beelen R.H. (1996). Ingestion of Staphylococcus Aureus, Staphylococcus Epidermidis, and Escherichia Coli by Human Peritoneal Mesothelial Cells. Infect. Immun..

[26] Wagner B.J., Lindau D., Ripper D., Stierhof Y.D., Glatzle J., Witte M., Beck H., Keppeler H., Lauber K., Rammensee H.G. (2011). Phagocytosis of Dying Tumor Cells by Human Peritoneal Mesothelial Cells. J. Cell Sci..

[27] Shaw T.J., Zhang X.Y., Huo Z., Robertson D., Lovell P.A., Dalgleish A.G., Barton D.P.J. (2016). Human Peritoneal Mesothelial Cells Display Phagocytic and Antigen-Presenting Functions to Contribute to Intraperitoneal Immunity. Int. J. Gynecol. Cancer.

[28] Hausmann M.J., Rogachev B., Weiler M., Chaimovitz C., Douvdevani A. (2000). Accessory Role of Human Peritoneal Mesothelial Cells in Antigen Presentation and T-Cell Growth. Kidney Int..

[29] Rennard S.I., Jaurand M.C., Bignon J., Kawanami O., Ferrans V.J., Davidson J., Crystal R.G. (1984). Role of Pleural Mesothelial Cells in the Production of the Submesothelial Connective Tissue Matrix of Lung. Am. Rev. Respir. Dis..

[30] Ma C., Tarnuzzer R.W., Chegini N. (1999). Expression of Matrix Metalloproteinases and Tissue Inhibitor of Matrix Metalloproteinases in Mesothelial Cells and Their Regulation by Transforming Growth Factor-β1. Wound Repair. Regen..

[31] Xiao L., Sun L., Liu F., Peng Y., Duan S. (2010). Connective Tissue Growth Factor Knockdown Attenuated Matrix Protein Production and Vascular Endothelial Growth Factor Expression Induced by Transforming Growth Factor-β1 in Cultured Human Peritoneal Mesothelial Cells. Ther. Apher. Dial..

[32] López-Cabrera M. (2014). Mesenchymal Conversion of Mesothelial Cells Is a Key Event in the Pathophysiology of the Peritoneum during Peritoneal Dialysis. Adv. Med..

[33] Yáñez-Mó M., Siljander P.R.-M., Andreu Z., Bedina Zavec A., Borràs F.E., Buzas E.I., Buzas K., Casal E., Cappello F., Carvalho J. (2015). Biological Properties of Extracellular Vesicles and Their Physiological Functions. J. Extracell. Vesicles.

[34] Namvar S., Woolf A.S., Zeef L.A., Wilm T., Wilm B., Herrick S.E. (2018). Functional Molecules in Mesothelial-to-mesenchymal Transition Revealed by Transcriptome Analyses. J. Pathol..

[35] Ramundo V., Zanirato G., Aldieri E. (2021). The Epithelial-to-Mesenchymal Transition (EMT) in the Development and Metastasis of Malignant Pleural Mesothelioma. Int. J. Mol. Sci..

[36] Zeisberg M., Hanai J., Sugimoto H., Mammoto T., Charytan D., Strutz F., Kalluri R. (2003). BMP-7 Counteracts TGF-Β1–Induced Epithelial-to-Mesenchymal Transition and Reverses Chronic Renal Injury. Nat. Med..

[37] Nasreen N., Mohammed K.A., Mubarak K.K., Baz M.A., Akindipe O.A., Fernandez-Bussy S., Antony V.B. (2009). Pleural Mesothelial Cell Transformation into Myofibroblasts and Haptotactic Migration in Response to TGF-Β1 in Vitro. Am. J. Physiol.-Lung Cell. Mol. Physiol..

[38] Pérez-Lozano M.L., Sandoval P., Rynne-Vidal Á., Aguilera A., Jiménez-Heffernan J.A., Albar-Vizcaíno P., Majano P.L., Sánchez-Tomero J.A., Selgas R., López-Cabrera M. (2013). Functional Relevance of the Switch of VEGF Receptors/Co-Receptors during Peritoneal Dialysis-Induced Mesothelial to Mesenchymal Transition. PLoS ONE.

[39] Aashaq S., Batool A., Mir S.A., Beigh M.A., Andrabi K.I., Shah Z.A. (2022). TGF-β Signaling: A Recap of SMAD-independent and SMAD-dependent Pathways. J. Cell Physiol..

[40] Debnath P., Huirem R.S., Dutta P., Palchaudhuri S. (2022). Epithelial–Mesenchymal Transition and Its Transcription Factors. Biosci. Rep..

[41] Lee J.H., Massagué J. (2022). TGF-β in Developmental and Fibrogenic EMTs. Semin. Cancer Biol..

[42] Strippoli R., Moreno-Vicente R., Battistelli C., Cicchini C., Noce V., Amicone L., Marchetti A., del Pozo M.A., Tripodi M. (2016). Molecular Mechanisms Underlying Peritoneal EMT and Fibrosis. Stem Cells Int..

[43] Saitoh M. (2023). Transcriptional Regulation of EMT Transcription Factors in Cancer. Semin. Cancer Biol..

[44] Zhou J., Jain S., Azad A.K., Xu X., Yu H.C., Xu Z., Godbout R., Fu Y. (2016). Notch and TGFβ Form a Positive Regulatory Loop and Regulate EMT in Epithelial Ovarian Cancer Cells. Cell. Signal..

[45] Bakalenko N., Kuznetsova E., Malashicheva A. (2024). The Complex Interplay of TGF-β and Notch Signaling in the Pathogenesis of Fibrosis. Int. J. Mol. Sci..

[46] Wang J., Yang L., Mei J., Li Z., Huang Y., Sun H., Zheng K., Kuang H., Luo W. (2024). Knockdown of Notch Suppresses Epithelial-Mesenchymal Transition and Induces Angiogenesis in Oral Submucous Fibrosis by Regulating TGF-Β1. Biochem. Genet..

[47] Zhu F., Li T., Qiu F., Fan J., Zhou Q., Ding X., Nie J., Yu X. (2010). Preventive Effect of Notch Signaling Inhibition by a γ-Secretase Inhibitor on Peritoneal Dialysis Fluid-Induced Peritoneal Fibrosis in Rats. Am. J. Pathol..

[48] Ding C., Qin C., Sun Y., Liu Y., Tang G., Liao Z., Zhao C., Wu C., Wang L. (2025). Bioceramics-Enhanced Patch Activates Epicardial Epithelial-to-Mesenchymal Transition via Notch Pathway for Cardiac Repair. Sci. Adv..

[49] Zeisberg M., Kalluri R. (2004). The Role of Epithelial-to-Mesenchymal Transition in Renal Fibrosis. J. Mol. Med..

[50] Weiskirchen R. (2013). BMP-7 Counteracting TGF-Beta1 Activities in Organ Fibrosis. Front. Biosci..

[51] Piccolo S., Dupont S., Cordenonsi M. (2014). The Biology of YAP/TAZ: Hippo Signaling and Beyond. Physiol. Rev..

[52] Strippoli R., Sandoval P., Moreno-Vicente R., Rossi L., Battistelli C., Terri M., Pascual-Antón L., Loureiro M., Matteini F., Calvo E. (2020). Caveolin1 and YAP Drive Mechanically Induced Mesothelial to Mesenchymal Transition and Fibrosis. Cell Death Dis..

[53] Lua I., Balog S., Asahina K. (2022). TAZ/WWTR1 Mediates Liver Mesothelial–Mesenchymal Transition Induced by Stiff Extracellular Environment, TGF-β1, and Lysophosphatidic Acid. J. Cell Physiol..

[54] Koopmans T., Rinkevich Y. (2018). Mesothelial to Mesenchyme Transition as a Major Developmental and Pathological Player in Trunk Organs and Their Cavities. Commun. Biol..

[55] Liu L., Sun Q., Davis F., Mao J., Zhao H., Ma D. (2022). Epithelial–Mesenchymal Transition in Organ Fibrosis Development: Current Understanding and Treatment Strategies. Burn. Trauma.

[56] Que J., Wilm B., Hasegawa H., Wang F., Bader D., Hogan B.L.M. (2008). Mesothelium Contributes to Vascular Smooth Muscle and Mesenchyme during Lung Development. Proc. Natl. Acad. Sci. USA.

[57] Wilm B., Ipenberg A., Hastie N.D., Burch J.B.E., Bader D.M. (2005). The Serosal Mesothelium Is a Major Source of Smooth Muscle Cells of the Gut Vasculature. Development.

[58] Rinkevich Y., Mori T., Sahoo D., Xu P.X., Bermingham J.R., Weissman I.L. (2012). Identification and Prospective Isolation of a Mesothelial Precursor Lineage Giving Rise to Smooth Muscle Cells and Fibroblasts for Mammalian Internal Organs, and Their Vasculature. Nat. Cell Biol..

[59] Braitsch C.M., Combs M.D., Quaggin S.E., Yutzey K.E. (2012). Pod1/Tcf21 Is Regulated by Retinoic Acid Signaling and Inhibits Differentiation of Epicardium-Derived Cells into Smooth Muscle in the Developing Heart. Dev. Biol..

[60] van Tuyn J., Atsma D.E., Winter E.M., van der Velde-van Dijke I., Pijnappels D.A., Bax N.A.M., Knaän-Shanzer S., Gittenberger-de Groot A.C., Poelmann R.E., van der Laarse A. (2007). Epicardial Cells of Human Adults Can Undergo an Epithelial-to-Mesenchymal Transition and Obtain Characteristics of Smooth Muscle Cells In Vitro. Stem Cells.

[61] Lachaud C.C., López-Beas J., Soria B., Hmadcha A. (2014). EGF-Induced Adipose Tissue Mesothelial Cells Undergo Functional Vascular Smooth Muscle Differentiation. Cell Death Dis..

[62] Lachaud C.C., Pezzolla D., Domínguez-Rodríguez A., Smani T., Soria B., Hmadcha A. (2013). Functional Vascular Smooth Muscle-like Cells Derived from Adult Mouse Uterine Mesothelial Cells. PLoS ONE.

[63] Lansley S.M., Searles R.G., Hoi A., Thomas C., Moneta H., Herrick S.E., Thompson P.J., Mark N., Sterrett G.F., Prêle C.M. (2011). Mesothelial Cell Differentiation into Osteoblast- and Adipocyte-like Cells. J. Cell Mol. Med..

[64] Lua I., Asahina K. (2016). The Role of Mesothelial Cells in Liver Development, Injury, and Regeneration. Gut Liver.

[65] Mutsaers S.E., Whitaker D., Papadimitriou J.M. (2000). Mesothelial Regeneration Is Not Dependent on Subserosal Cells. J. Pathol..

[66] Shapiro L., Holste J.L., Muench T., diZerega G. (2015). Rapid Reperitonealization and Wound Healing in a Preclinical Model of Abdominal Trauma Repair with a Composite Mesh. Int. J. Surg..

[67] Foley-Comer A.J., Herrick S.E., Al-Mishlab T., Prêle C.M., Laurent G.J., Mutsaers S.E. (2002). Evidence for Incorporation of Free-Floating Mesothelial Cells as a Mechanism of Serosal Healing. J. Cell Sci..

[68] Jantz M.A., Antony V.B. (2006). Pleural Fibrosis. Clin. Chest Med..

[69] Raghu G., Chen S.-Y., Hou Q., Yeh W.-S., Collard H.R. (2016). Incidence and Prevalence of Idiopathic Pulmonary Fibrosis in US Adults 18–64 Years Old. Eur. Respir. J..

[70] Raghu G., Collard H.R., Egan J.J., Martinez F.J., Behr J., Brown K.K., Colby T.V., Cordier J.-F., Flaherty K.R., Lasky J.A. (2011). An Official ATS/ERS/JRS/ALAT Statement: Idiopathic Pulmonary Fibrosis: Evidence-Based Guidelines for Diagnosis and Management. Am. J. Respir. Crit. Care Med..

[71] Zolak J.S., Jagirdar R., Surolia R., Karki S., Oliva O., Hock T., Guroji P., Ding Q., Liu R.M., Bolisetty S. (2013). Pleural Mesothelial Cell Differentiation and Invasion in Fibrogenic Lung Injury. Am. J. Pathol..

[72] Yung S., Chan T.M. (2012). Pathophysiological Changes to the Peritoneal Membrane during PD-Related Peritonitis: The Role of Mesothelial Cells. Mediat. Inflamm..

[73] Zhao J. (2014). Molecular Mechanisms of AGE/RAGE-Mediated Fibrosis in the Diabetic Heart. World J. Diabetes.

[74] Fang M., Xiang F.L., Braitsch C.M., Yutzey K.E. (2016). Epicardium-Derived Fibroblasts in Heart Development and Disease. J. Mol. Cell Cardiol..

[75] Wynn T.A., Ramalingam T.R. (2012). Mechanisms of Fibrosis: Therapeutic Translation for Fibrotic Disease. Nat. Med..

[76] Wang R., Guo T., Li J. (2022). Mechanisms of Peritoneal Mesothelial Cells in Peritoneal Adhesion. Biomolecules.

[77] Marshall B.C., Santana A., Xu Q.P., Petersen M.J., Campbell E.J., Hoidal J.R., Welgus H.G. (1993). Metalloproteinases and Tissue Inhibitor of Metalloproteinases in Mesothelial Cells. Cellular Differentiation Influences Expression. J. Clin. Investig..

[78] Ishimura T., Ishii A., Yamada H., Osaki K., Toda N., Mori K.P., Ohno S., Kato Y., Handa T., Sugioka S. (2023). Matrix Metalloproteinase-10 Deficiency Has Protective Effects against Peritoneal Inflammation and Fibrosis via Transcription Factor NFκΒ Pathway Inhibition. Kidney Int..

[79] Merkle M., Ribeiro A., Sauter M., Ladurner R., Mussack T., Sitter T., Wörnle M. (2010). Effect of Activation of Viral Receptors on the Gelatinases MMP-2 and MMP-9 in Human Mesothelial Cells. Matrix Biol..

[80] Fukudome K., Fujimoto S., Sato Y., Hisanaga S., Eto T. (2001). Peritonitis Increases MMP-9 Activity in Peritoneal Effluent from CAPD Patients. Nephron.

[81] Bajaj M.S., Pendurthi U., Koenig K., Pueblitz S., Idell S. (2000). Tissue Factor Pathway Inhibitor Expression by Human Pleural Mesothelial and Mesothelioma Cells. Eur. Respir. J..

[82] Idell S., Zwieb C., Kumar A., Koenig K.B., Johnson A.R. (1992). Pathways of Fibrin Turnover of Human Pleural Mesothelial Cells In Vitro. Am. J. Respir. Cell Mol. Biol..

[83] Ivarsson M.L., Holmdahl L., Falk P., Mölne J., Risberg B. (1998). Characterization and Fibrinolytic Properties of Mesothelial Cells Isolated from Peritoneal Lavage. Scand. J. Clin. Lab. Investig..

[84] Koninckx P.R., Gomel V., Ussia A., Adamyan L. (2016). Role of the Peritoneal Cavity in the Prevention of Postoperative Adhesions, Pain, and Fatigue. Fertil. Steril..

[85] Honjo K., Munakata S., Tashiro Y., Salama Y., Shimazu H., Eiamboonsert S., Dhahri D., Ichimura A., Dan T., Miyata T. (2017). Plasminogen Activator Inhibitor-1 Regulates Macrophage-dependent Postoperative Adhesion by Enhancing EGF-HER1 Signaling in Mice. FASEB J..

[86] Wight T.N., Potter-Perigo S. (2011). The Extracellular Matrix: An Active or Passive Player in Fibrosis?. Am. J. Physiol.-Gastrointest. Liver Physiol..

[87] Baum J., Duffy H.S. (2011). Fibroblasts and Myofibroblasts: What Are We Talking About?. J. Cardiovasc. Pharmacol..

[88] Younesi F.S., Miller A.E., Barker T.H., Rossi F.M.V., Hinz B. (2024). Fibroblast and Myofibroblast Activation in Normal Tissue Repair and Fibrosis. Nat. Rev. Mol. Cell Biol..

[89] Yang A.H., Chen J.Y., Lin J.K. (2003). Myofibroblastic Conversion of Mesothelial Cells. Kidney Int..

[90] Brønnum H., Andersen D.C., Schneider M., Yaël Nossent A., Nielsen S.B., Sheikh S.P. (2013). Islet-1 Is a Dual Regulator of Fibrogenic Epithelial-to-Mesenchymal Transition in Epicardial Mesothelial Cells. Exp. Cell Res..

[91] Liu F., Yu F., Lu Y.Z., Cheng P.P., Liang L.M., Wang M., Chen S.J., Huang Y., Song L.J., He X.L. (2020). Crosstalk between Pleural Mesothelial Cell and Lung Fibroblast Contributes to Pulmonary Fibrosis. Biochim. Biophys. Acta (BBA)—Mol. Cell Res..

[92] Lua I., Li Y., Pappoe L.S., Asahina K. (2015). Myofibroblastic Conversion and Regeneration of Mesothelial Cells in Peritoneal and Liver Fibrosis. Am. J. Pathol..

[93] Wilm T.P., Tanton H., Mutter F., Foisor V., Middlehurst B., Ward K., Benameur T., Hastie N., Wilm B. (2021). Restricted Differentiative Capacity of Wt1-Expressing Peritoneal Mesothelium in Postnatal and Adult Mice. Sci. Rep..

[94] Sontake V., Kasam R.K., Sinner D., Korfhagen T.R., Reddy G.B., White E.S., Jegga A.G., Madala S.K. (2018). Wilms’ Tumor 1 Drives Fibroproliferation and Myofibroblast Transformation in Severe Fibrotic Lung Disease. JCI Insight.

[95] Kadri S., Fischer A., Mück-Häusl M., Han W., Kadri A., Lin Y., Yang L., Hu S., Ye H., Ramesh P. (2025). A Mesothelial Differentiation Gateway Drives Fibrosis. Nat. Commun..

[96] Kis K., Liu X., Hagood J.S. (2011). Myofibroblast Differentiation and Survival in Fibrotic Disease. Expert Rev. Mol. Med..

[97] Kramann R., DiRocco D.P., Humphreys B.D. (2013). Understanding the Origin, Activation and Regulation of Matrix-Producing Myofibroblasts for Treatment of Fibrotic Disease. J. Pathol..

[98] Micallef L., Vedrenne N., Billet F., Coulomb B., Darby I.A., Desmoulière A. (2012). The Myofibroblast, Multiple Origins for Major Roles in Normal and Pathological Tissue Repair. Fibrogenesis Tissue Repair.

[99] Homps-Legrand M., Crestani B., Mailleux A.A. (2023). Origins of Pathological Myofibroblasts in Lung Fibrosis: Insights from Lineage Tracing Mouse Models in the Single-Cell RNA Sequencing Era. Am. J. Physiol.-Lung Cell. Mol. Physiol..

[100] Meng X., Nikolic-Paterson D.J., Lan H.Y. (2016). TGF-β: The Master Regulator of Fibrosis. Nat. Rev. Nephrol..

[101] Xie C., Huang J.Q., Light R.W. (2005). The Effects of Erythromycin on the Viability and the Secretion of TNF-α and TGF-β1 and Expression of Connexin43 by Human Pleural Mesothelial Cells. Respirology.

[102] Saed G.M., Kruger M., Diamond M.P. (2004). Expression of Transforming Growth Factor-β and Extracellular Matrix by Human Peritoneal Mesothelial Cells and by Fibroblasts from Normal Peritoneum and Adhesions: Effect of Tisseel. Wound Repair Regen..

[103] Saed G.M., Zhang W., Diamond M.P., Chegini N., Holmdahl L. (2000). Transforming Growth Factor Beta Isoforms Production by Human Peritoneal Mesothelial Cells after Exposure to Hypoxia. Am. J. Reprod. Immunol..

[104] Antony V.B., Sahn S.A., Mossman B., Gail D.B., Kalica A. (1992). Pleural Cell Biology in Health and Disease. Am. Rev. Respir. Dis..

[105] Yue X., Shan B., Lasky J.A. (2010). TGF-β: Titan of Lung Fibrogenesis. Curr. Enzym. Inhib..

[106] Vallée A., Lecarpentier Y. (2019). TGF-β in Fibrosis by Acting as a Conductor for Contractile Properties of Myofibroblasts. Cell Biosci..

[107] Biernacka A., Dobaczewski M., Frangogiannis N.G. (2011). TGF-β Signaling in Fibrosis. Growth Factors.

[108] Ross R., Raines E.W., Bowen-Pope D.F. (1986). The Biology of Platelet-Derived Growth Factor. Cell.

[109] Pierce G.F., Mustoe T.A., Altrock B.W., Deuel T.F., Thomason A. (1991). Role of Platelet-derived Growth Factor in Wound Healing. J. Cell Biochem..

[110] Chen Y.T., Chang Y.T., Pan S.Y., Chou Y.H., Chang F.C., Yeh P.Y., Liu Y.H., Chiang W.C., Chen Y.M., Wu K.D. (2014). Lineage Tracing Reveals Distinctive Fates for Mesothelial Cells and Submesothelial Fibroblasts during Peritoneal Injury. J. Am. Soc. Nephrol..

[111] Adamson I.Y., Prieditis H., Young L. (1997). Lung Mesothelial Cell and Fibroblast Responses to Pleural and Alveolar Macrophage Supernatants and to Lavage Fluids from Crocidolite-Exposed Rats. Am. J. Respir. Cell Mol. Biol..

[112] Pierce G.F., Mustoe T.A., Lingelbach J., Masakowski V.R., Griffin G.L., Senior R.M., Deuel T.F. (1989). Platelet-Derived Growth Factor and Transforming Growth Factor-Beta Enhance Tissue Repair Activities by Unique Mechanisms. J. Cell Biol..

[113] Antony V.B., Nasreen N., Mohammed K.A., Sriram P.S., Frank W., Schoenfeld N., Loddenkemper R. (2004). Talc Pleurodesis. Chest.

[114] Wei Y.S., Tsai S.Y., Lin S.L., Chen Y.T., Tsai P.S. (2025). Methylglyoxal-Stimulated Mesothelial Cells Prompted Fibroblast-to-Proto-Myofibroblast Transition. Int. J. Mol. Sci..

[115] Lv L.L., Feng Y., Wu M., Wang B., Li Z.L., Zhong X., Wu W.J., Chen J., Ni H.F., Tang T.T. (2020). Exosomal MiRNA-19b-3p of Tubular Epithelial Cells Promotes M1 Macrophage Activation in Kidney Injury. Cell Death Differ..

[116] Yu M., Shi J., Sheng M. (2018). Exosomes: The New Mediator of Peritoneal Membrane Function. Kidney Blood Press. Res..

[117] Huang Q., Sun Y., Peng L., Sun J., Sha Z., Lin H., Li Y., Li C., Li H., Shang H. (2023). Extracellular Vesicle-packaged ILK from Mesothelial Cells Promotes Fibroblast Activation in Peritoneal Fibrosis. J. Extracell. Vesicles.

[118] Parker M.W., Rossi D., Peterson M., Smith K., Sikström K., White E.S., Connett J.E., Henke C.A., Larsson O., Bitterman P.B. (2014). Fibrotic Extracellular Matrix Activates a Profibrotic Positive Feedback Loop. J. Clin. Investig..

[119] Herrera J., Henke C.A., Bitterman P.B. (2018). Extracellular Matrix as a Driver of Progressive Fibrosis. J. Clin. Investig..

[120] Su H., Zou R., Su J., Chen X., Yang H., An N., Yang C., Tang J., Liu H., Yao C. (2024). Sterile Inflammation of Peritoneal Membrane Caused by Peritoneal Dialysis: Focus on the Communication between Immune Cells and Peritoneal Stroma. Front. Immunol..

[121] Lai K.N., Leung J.C.K. (2010). Inflammation in Peritoneal Dialysis. Nephron Clin. Pract..

[122] Helmke A., Nordlohne J., Balzer M.S., Dong L., Rong S., Hiss M., Shushakova N., Haller H., von Vietinghoff S. (2019). CX3CL1–CX3CR1 Interaction Mediates Macrophage-Mesothelial Cross Talk and Promotes Peritoneal Fibrosis. Kidney Int..

[123] Yao V., Platell C., Hall J.C. (2004). Peritoneal Mesothelial Cells Produce Inflammatory Related Cytokines. ANZ J. Surg..

[124] van Grevenstein W.M.U., Hofland L.J., van Rossen M.E.E., van Koetsveld P.M., Jeekel J., van Eijck C.H.J. (2007). Inflammatory Cytokines Stimulate the Adhesion of Colon Carcinoma Cells to Mesothelial Monolayers. Dig. Dis. Sci..

[125] Anders H.J., Schaefer L. (2014). Beyond Tissue Injury—Damage-Associated Molecular Patterns, Toll-Like Receptors, and Inflammasomes Also Drive Regeneration and Fibrosis. J. Am. Soc. Nephrol..

[126] Raby A.C., González-Mateo G.T., Williams A., Topley N., Fraser D., López-Cabrera M., Labéta M.O. (2018). Targeting Toll-like Receptors with Soluble Toll-like Receptor 2 Prevents Peritoneal Dialysis Solution–Induced Fibrosis. Kidney Int..

[127] Chu Y., Wang Y., Zheng Z., Lin Y., He R., Liu J., Yang X. (2017). Proinflammatory Effect of High Glucose Concentrations on HMrSV5 Cells via the Autocrine Effect of HMGB1. Front. Physiol..

[128] Nevado J., Peiró C., Vallejo S., El-Assar M., Lafuente N., Matesanz N., Azcutia V., Cercas E., Sánchez-Ferrer C.F., Rodríguez-Mañas L. (2005). Amadori Adducts Activate Nuclear Factor-*κ* B-related Proinflammatory Genes in Cultured Human Peritoneal Mesothelial Cells. Br. J. Pharmacol..

[129] Matsuo H., Tamura M., Kabashima N., Serino R., Tokunaga M., Shibata T., Matsumoto M., Aijima M., Oikawa S., Anai H. (2006). Prednisolone Inhibits Hyperosmolarity-Induced Expression of MCP-1 via NF-ΚB in Peritoneal Mesothelial Cells. Kidney Int..

[130] Dai T., Wang Y., Nayak A., Nast C.C., Quang L., LaPage J., Andalibi A., Adler S.G. (2014). Janus Kinase Signaling Activation Mediates Peritoneal Inflammation and Injury in Vitro and in Vivo in Response to Dialysate. Kidney Int..

[131] Park J.S., Kim Y.S., Jee Y.K., Myong N.H., Lee K.Y. (2003). Interleukin-8 Production in Tuberculous Pleurisy: Role of Mesothelial Cells Stimulated by Cytokine Network Involving Tumour Necrosis Factor-α and Interleukin-1β. Scand. J. Immunol..

[132] Mierzejewski M., Paplinska-Goryca M., Korczynski P., Krenke R. (2021). Primary Human Mesothelial Cell Culture in the Evaluation of the Inflammatory Response to Different Sclerosing Agents Used for Pleurodesis. Physiol. Rep..

[133] Scheller J., Ohnesorge N., Rose-John S. (2006). Interleukin-6 Trans-Signalling in Chronic Inflammation and Cancer. Scand. J. Immunol..

[134] Le T.T.T., Karmouty-Quintana H., Melicoff E., Le T.T.T., Weng T., Chen N.-Y., Pedroza M., Zhou Y., Davies J., Philip K. (2014). Blockade of IL-6 Trans Signaling Attenuates Pulmonary Fibrosis. J. Immunol..

[135] Capobianco A., Cottone L., Monno A., Manfredi A.A., Rovere-Querini P. (2017). The Peritoneum: Healing, Immunity, and Diseases. J. Pathol..

[136] Delo J., Forton D., Triantafyllou E., Singanayagam A. (2023). Peritoneal Immunity in Liver Disease. Livers.

[137] Ivanov S., Gallerand A., Gros M., Stunault M.I., Merlin J., Vaillant N., Yvan-Charvet L., Guinamard R.R. (2019). Mesothelial Cell CSF1 Sustains Peritoneal Macrophage Proliferation. Eur. J. Immunol..

[138] Lai C.W., Bagadia P., Barisas D.A.G., Jarjour N.N., Wong R., Ohara T., Muegge B.D., Lu Q., Xiong S., Edelson B.T. (2022). Mesothelium-Derived Factors Shape GATA6-Positive Large Cavity Macrophages. J. Immunol..

[139] Wang J., Kubes P. (2016). A Reservoir of Mature Cavity Macrophages That Can Rapidly Invade Visceral Organs to Affect Tissue Repair. Cell.

[140] Amari M., Taguchi K., Iwahara M., Oharaseki T., Yokouchi Y., Naoe S., Takahashi K. (2006). Interaction between Mesothelial Cells and Macrophages in the Initial Process of Pleural Adhesion: Ultrastructural Studies Using Adhesion Molecules. Med. Mol. Morphol..

[141] Nasreen N., Mohammed K.A., Ward M.J., Antony V.B. (1999). Mycobacterium-Induced Transmesothelial Migration of Monocytes into Pleural Space: Role of Intercellular Adhesion Molecule–1 in Tuberculous Pleurisy. J. Infect. Dis..

[142] Bellingan G.J., Xu P., Cooksley H., Cauldwell H., Shock A., Bottoms S., Haslett C., Mutsaers S.E., Laurent G.J. (2002). Adhesion Molecule–Dependent Mechanisms Regulate the Rate of Macrophage Clearance During the Resolution of Peritoneal Inflammation. J. Exp. Med..

[143] Suarez J.S., Novelli F., Goto K., Ehara M., Steele M., Kim J.H., Zolondick A.A., Xue J., Xu R., Saito M. (2023). HMGB1 Released by Mesothelial Cells Drives the Development of Asbestos-Induced Mesothelioma. Proc. Natl. Acad. Sci. USA.

[144] Baumann M.H., Heinrich K., Sahn S.A., Strange C. (1993). Pleural Macrophages Differentially Alter Mesothelial Cell Growth and Collagen Production. Inflammation.

[145] Shi J., Li Q., Sheng M., Zheng M., Yu M., Zhang L. (2016). The Role of TLR4 in M1 Macrophage-Induced Epithelial-Mesenchymal Transition of Peritoneal Mesothelial Cells. Cell. Physiol. Biochem..

[146] Tian L., Yu Q., Liu D., Chen Z., Zhang Y., Lu J., Ma X., Huang F., Han J., Wei L. (2022). Epithelial–Mesenchymal Transition of Peritoneal Mesothelial Cells Is Enhanced by M2c Macrophage Polarization. Immunol. Investig..

[147] Katz S., Balogh P., Kiss A.L. (2011). Mesothelial Cells Can Detach from the Mesentery and Differentiate into Macrophage-like Cells. APMIS.

[148] Katz S., Balogh P., Nagy N., Kiss A.L. (2012). Epithelial-To-Mesenchymal Transition Induced by Freund’s Adjuvant Treatment in Rat Mesothelial Cells: A Morphological and Immunocytochemical Study. Pathol. Oncol. Res..

[149] Katz S., Zsiros V., Dóczi N., Szabó A., Biczó Á., Kiss A.L. (2016). GM-CSF and GM-CSF Receptor Have Regulatory Role in Transforming Rat Mesenteric Mesothelial Cells into Macrophage-like Cells. Inflamm. Res..

[150] Zsiros V., Kiss A.L. (2020). Cellular and Molecular Events of Inflammation Induced Transdifferentiation (EMT) and Regeneration (MET) in Mesenteric Mesothelial Cells. Inflamm. Res..

[151] Yuan M.L., Tong Z.H., Jin X.G., Zhang J.C., Wang X.J., Ma W.L., Yin W., Zhou Q., Ye H., Shi H.Z. (2013). Regulation of CD4+ T Cells by Pleural Mesothelial Cells via Adhesion Molecule-Dependent Mechanisms in Tuberculous Pleurisy. PLoS ONE.

[152] Witowski J., Ksiązek K., Warnecke C., Kuźlan M., Korybalska K., Tayama H., Wiśniewska-Elnur J., Pawlaczyk K., Trómińska J., Bręborowicz A. (2007). Role of Mesothelial Cell-Derived Granulocyte Colony-Stimulating Factor in Interleukin-17-Induced Neutrophil Accumulation in the Peritoneum. Kidney Int..

[153] Witowski J., Pawlaczyk K., Breborowicz A., Scheuren A., Kuzlan-Pawlaczyk M., Wisniewska J., Polubinska A., Friess H., Gahl G.M., Frei U. (2000). IL-17 Stimulates Intraperitoneal Neutrophil Infiltration Through the Release of GROα Chemokine from Mesothelial Cells. J. Immunol..

[154] Bettelli E., Carrier Y., Gao W., Korn T., Strom T.B., Oukka M., Weiner H.L., Kuchroo V.K. (2006). Reciprocal Developmental Pathways for the Generation of Pathogenic Effector TH17 and Regulatory T Cells. Nature.

[155] Littman D.R., Rudensky A.Y. (2010). Th17 and Regulatory T Cells in Mediating and Restraining Inflammation. Cell.

[156] Zhou L., Lopes J.E., Chong M.M.W., Ivanov I.I., Min R., Victora G.D., Shen Y., Du J., Rubtsov Y.P., Rudensky A.Y. (2008). TGF-β-Induced Foxp3 Inhibits TH17 Cell Differentiation by Antagonizing RORγt Function. Nature.

[157] Korn T., Anderson A.C., Bettelli E., Oukka M. (2007). The Dynamics of Effector T Cells and Foxp3+ Regulatory T Cells in the Promotion and Regulation of Autoimmune Encephalomyelitis. J. Neuroimmunol..

[158] Liappas G., Gónzalez-Mateo G.T., Majano P., Sánchez- Tomero J.A., Ruiz-Ortega M., Rodrigues Díez R., Martín P., Sanchez-Díaz R., Selgas R., López-Cabrera M. (2015). T Helper 17/Regulatory T Cell Balance and Experimental Models of Peritoneal Dialysis-Induced Damage. Biomed. Res. Int..

[159] Helmke A., Hüsing A.M., Gaedcke S., Brauns N., Balzer M.S., Reinhardt M., Hiss M., Shushakova N., de Luca D., Prinz I. (2021). Peritoneal Dialysate-range Hypertonic Glucose Promotes T-cell IL-17 Production That Induces Mesothelial Inflammation. Eur. J. Immunol..

[160] Gauthier B.R., Rubio-Contreras D., Gómez-Rosado J.C., Capitán-Morales L.C., Hmadcha A., Soria B., Lachaud C.C. (2022). Human Omental Mesothelial Cells Impart an Immunomodulatory Landscape Impeding B- and T-Cell Activation. Int. J. Mol. Sci..

[161] Lin C.Y., Kift-Morgan A., Moser B., Topley N., Eberl M. (2013). Suppression of Pro-Inflammatory T-Cell Responses by Human Mesothelial Cells. Nephrol. Dial. Transplant..

[162] Kitayama J., Emoto S., Yamaguchi H., Ishigami H., Yamashita H., Seto Y., Matsuzaki K., Watanabe T. (2014). CD90(+)CD45(−) Intraperitoneal Mesothelial-like Cells Inhibit T Cell Activation by Production of Arginase I. Cell Immunol..

[163] Ramjee V., Li D., Manderfield L.J., Liu F., Engleka K.A., Aghajanian H., Rodell C.B., Lu W., Ho V., Wang T. (2017). Epicardial YAP/TAZ Orchestrate an Immunosuppressive Response Following Myocardial Infarction. J. Clin. Investig..

[164] Klein C.L., Bittinger F., Skarke C.C., Wagner M., Köhler H., Walgenbach S., Kirkpatrick J. (1995). Effects of Cytokines on the Expression of Cell Adhesion Molecules by Cultured Human Omental Mesothelial Cells. Pathobiology.

[165] Terri M., Trionfetti F., Montaldo C., Cordani M., Tripodi M., Lopez-Cabrera M., Strippoli R. (2021). Mechanisms of Peritoneal Fibrosis: Focus on Immune Cells–Peritoneal Stroma Interactions. Front. Immunol..

[166] Sacnun J.M., Herzog R., Kratochwill K. (2022). Proteomic Study of Mesothelial and Endothelial Cross-Talk: Key Lessons. Expert. Rev. Proteom..

[167] Ito Y., Sun T., Tawada M., Kinashi H., Yamaguchi M., Katsuno T., Kim H., Mizuno M., Ishimoto T. (2024). Pathophysiological Mechanisms of Peritoneal Fibrosis and Peritoneal Membrane Dysfunction in Peritoneal Dialysis. Int. J. Mol. Sci..

[168] Hanumegowda C., Farkas L., Kolb M. (2012). Angiogenesis in Pulmonary Fibrosis. Chest.

[169] Zhu N., Gu L., Jia J., Wang X., Wang L., Yang M., Yuan W. (2019). Endothelin-1 Triggers Human Peritoneal Mesothelial Cells’ Proliferation via ERK1/2-Ets-1 Signaling Pathway and Contributes to Endothelial Cell Angiogenesis. J. Cell Biochem..

[170] Kinashi H., Ito Y., Sun T., Katsuno T., Takei Y. (2018). Roles of the TGF-β–VEGF-C Pathway in Fibrosis-Related Lymphangiogenesis. Int. J. Mol. Sci..

[171] Bates D.O. (2010). Vascular Endothelial Growth Factors and Vascular Permeability. Cardiovasc. Res..

[172] Shibuya M. (2013). Vascular Endothelial Growth Factor and Its Receptor System: Physiological Functions in Angiogenesis and Pathological Roles in Various Diseases. J. Biochem..

[173] Boulanger E., Grossin N., Wautier M.P., Taamma R., Wautier J.L. (2007). Mesothelial RAGE Activation by AGEs Enhances VEGF Release and Potentiates Capillary Tube Formation. Kidney Int..

[174] Béguin E.P., van den Eshof B.L., Hoogendijk A.J., Nota B., Mertens K., Meijer A.B., van den Biggelaar M. (2019). Integrated Proteomic Analysis of Tumor Necrosis Factor α and Interleukin 1β-Induced Endothelial Inflammation. J. Proteom..

[175] Gerhardt T., Ley K. (2015). Monocyte Trafficking across the Vessel Wall. Cardiovasc. Res..

[176] Medrano-Bosch M., Simón-Codina B., Jiménez W., Edelman E.R., Melgar-Lesmes P. (2023). Monocyte-Endothelial Cell Interactions in Vascular and Tissue Remodeling. Front. Immunol..

[177] Das K., Qin W., Jeffers A., Owens S., Destarac L., Idell S., Rao L.V.M., Tucker T.A., Keshava S. (2025). Extracellular Vesicles Contribute to the Pathophysiology and Progression of Pleural Fibrosis by Promoting Mesothelial-to-Mesenchymal Transition and Neoangiogenesis. Am. J. Respir. Cell Mol. Biol..

[178] Aoki S., Takezawa T., Oshikata-Miyazaki A., Ikeda S., Kuroyama H., Chimuro T., Oguchi Y., Noguchi M., Narisawa Y., Toda S. (2014). Epithelial-to-Mesenchymal Transition and Slit Function of Mesothelial Cells Are Regulated by the Cross Talk between Mesothelial Cells and Endothelial Cells. Am. J. Physiol.-Ren. Physiol..

